# Genome Divergence Based on Entropic Segmentation of DNA

**DOI:** 10.3390/e27101019

**Published:** 2025-09-28

**Authors:** Pedro A. Bernaola-Galván, Pedro Carpena, Cristina Gómez-Martín, José L. Oliver

**Affiliations:** 1 Department of Applied Physics II, University of Málaga, 29071 Málaga, Spain; pcarpena@ctima.uma.es; 2 Institute Carlos I for Theoretical and Computational Physics, University of Málaga, 29071 Málaga, Spain; 3 Department of Genetics, Faculty of Sciences, University of Granada, 18071 Granada, Spain; c.a.gomezmartin@amsterdamumc.nl (C.G.-M.); oliver@ugr.es (J.L.O.); 4 Laboratory of Bioinformatics, Institute of Biotechnology, Center of Biomedical Research, University of Granada, 18100 Granada, Spain; 5 Department of Pathology, Cancer Center Amsterdam, Amsterdam University Medical Center, Vrije Universiteit Amsterdam, 1081 HV Amsterdam, The Netherlands

**Keywords:** entropic segmentation, Jensen-Shannon divergence, genome signatures, comparative genomics, genome compositional evolution, large-scale evolutionary patterns

## Abstract

The concept of a genome signature broadly refers to characteristic patterns in DNA sequences that enable the identification and comparison of species or individuals, often without requiring sequence alignment. Such signatures have applications ranging from forensic identification of individuals to cancer genomics. In comparative genomics and evolutionary biology, genome signatures typically rely on statistical properties of DNA that are species-specific and carry phylogenetic information reflecting evolutionary relationships. We propose a novel genome signature based on the compositional structure of DNA, defined by the distributions of strong/weak, purine/pyrimidine, and keto/amino ratios across DNA segments identified through entropic segmentation. We observe that these ratio distributions are similar among closely related species but differ markedly between distant ones. To quantify these differences, we employ the Jensen–Shannon distance—a symmetric and robust measure of distributional dissimilarity—to define a genome-to-genome distance metric, termed Segment Compositional Distance (D). Our results demonstrate a clear correlation between D and species divergence times, and also that this metric captures a strong phylogenetic signal. Our method employs a genome-wide approach rather than tracking specific mutations; thus, D offers a coarse-grained perspective on genome compositional evolution, contributing to the ongoing discussion surrounding the molecular clock hypothesis.

## 1. Introduction

Genome signatures refer to unique, identifiable patterns found within the sequence of nucleotides (DNA or RNA) that are characteristic of a particular species or organism. The concept of genome signatures was first introduced by Karlin et al. [1], who observed that certain nucleotide patterns could serve as markers for distinguishing different genomes. In general, these signatures can be derived not only from nucleotide composition [1] but also from various genomic features, such as *k*-mer frequencies (subsequences of length *k* within a genome) [2,3], codon usage [4], and sequence motifs [5]. Genomic signatures have been shown to possess significant biological relevance, capturing more than just compositional statistics. For example, Dick et al. [6] emphasizes how patterns such as oligonucleotide usage reflect evolutionary and functional processes within genomes. Galperin [7] further highlights how these signatures are shaped by selective pressures and genomic organization, providing a link between compositional features and biological function. Together, these studies underscore the value of genome signatures as meaningful descriptors of the evolutionary and structural characteristics of genomes.

The study of genome signatures has become an invaluable tool for a variety of biological applications, including phylogenetic analysis [2,8], microbial taxonomy [9,10], and functional genomics [1,4]. By examining the genome signatures of organisms, researchers can infer evolutionary relationships [2], identify new microorganisms [10], and predict gene functions based on conserved sequence patterns [1]. As genomic technologies and computational methods continue to advance, the use of genome signatures is expected to play an increasingly prominent role in understanding genomic diversity and complexity across the tree of life [8,11].

Differences in genomic signatures have long been employed as a basis for measuring evolutionary divergence between species and have been used to quantify genomic distances and infer phylogenetic relationships [2,9,10]. Such approaches are particularly valuable when traditional alignment-based methods are infeasible due to high sequence divergence or genome rearrangements. As a result, alignment-free methods that exploit genome signatures have gained popularity in comparative genomics and microbial taxonomy for assessing evolutionary relatedness across a wide range of organisms [8,11].

Most genome signatures proposed to date rely on global properties of the genome, such as overall nucleotide or *k*-mer composition, or motif distribution patterns (see [12] for a recent review). In contrast, we propose a novel type of genome signature that captures the large-scale structure of genomic heterogeneity. To achieve this, we first segment the genome into compositionally homogeneous regions [13,14], thereby uncovering its underlying compositional organization. Once the sequence is partitioned into these homogeneous segments, using the Jensen–Shannon divergence as a measure of heterogeneity [15], we construct the signature by computing statistical descriptors based on their properties—such as length, composition, and distribution—providing a more structured and spatially informed characterization of the genome. As we will show later, histograms of nucleotide compositional biases across genomic segments emerge as particularly effective candidates for species-specific signatures, showing marked differences between distantly related organisms. To quantify the dissimilarity between these histograms, we use the Jensen–Shannon distance—a symmetric, robust, and mathematically sound metric derived from the Jensen–Shannon divergence. This approach captures large-scale compositional variation in genomes and provides a meaningful, alignment-free method for assessing species divergence.

The remainder of the paper is structured as follows: In Section 2, we describe the procedure for segmenting DNA sequences and discuss the suitability of the heuristic algorithm proposed in [13]. Section 3 introduces the compositional landscape (This term is used here to describe the distribution of compositional features along the genome, analogous to the genomic landscape which refers to the distribution of genes or other genomic elements.) based on histograms of segment composition. In Section 4, we define an entropic distance measure between compositional landscapes—Segment Compositional Distance—which serves as a metric for quantifying compositional divergence between species. Section 5 examines the correlation between Segment Compositional Distance and species divergence times, demonstrates its phylogenetic signal, and explores its application in phylogenetic tree reconstruction. Finally, Section 6 and Section 7 summarize and discuss the findings of the paper.

## 2. DNA Sequence Segmentation

Due to the widespread spatial variability in nucleotide composition observed in most genomes [16], identifying compositionally homogeneous regions within DNA sequences is essential for understanding genomic architecture [17]. This task is fundamental in computational molecular biology [18], as it enables researchers to explore the large-scale organization of the genome content [19,20]. In simpler DNA sequences—such as those dominated by coding regions in prokaryotes, which lack long-range correlations—compositional domains can be readily identified [21]. However, in eukaryotic genomes characterized by complex long-range correlations and the absence of a typical patch length, the identification of such homogeneous segments becomes considerably more difficult [22,23]. To address this challenge, a statistical methodology that can estimate the locations of compositionally distinct boundaries with defined statistical confidence is required.

A widely used method is a heuristic, iterative segmentation algorithm [13,24,25], which partitions a DNA sequence, with a given statistical confidence, into non-overlapping, compositionally homogeneous domains. The main advantage of this algorithm is that it does not rely on any prior assumptions about segment size. In contrast, methods based on moving windows or fixed-size windows typically require additional analysis or filtering steps to account for window size limitations.

In brief, the segmentation algorithm can be described as follows:

1. Given a DNA sequence of length *N*, $S=\{b_{1},b_{2},\ldots ,b_{N}\}$, where $b_{i}\in \left\{A,C,T,G\right\}$, the algorithm slides a cursor along the sequence and computes at each position i=1,…,N−1 a divergence measure between the left $S_{1}=\left\{b_{1},b_{2},\ldots ,b_{i}\right\}$ and right $S_{2}=\left\{b_{i+1},\ldots ,b_{N}\right\}$ subsequences. The Jensen–Shannon divergence (JSD) is commonly used for this purpose because it is well-suited to symbolic data [14,15]:
  (1) $$d\left(i\right)=H\left(S\right)-\left[\frac{n_{1}}{N}H\left(S_{1}\right)+\frac{n_{2}}{N}H\left(S_{2}\right)\right],$$
  where $n_{1}=i$ and $n_{2}=N-i$ are the lengths of $S_{1}$ and $S_{2}$, respectively, *S* is the full sequence ($N=n_{1}+n_{2}$), and H(·) is the Shannon entropy of nucleotide frequencies:
  (2) $$H\left(S\right)=-\sum_{j\in \{A,C,T,G\}}f_{j}\log_{2}f_{j}.$$
  where $f_{j}$ is the frequency of nucleotide *j* in the corresponding subsequence.
2. Identify the position $i_{\max}$ that maximizes the divergence between the left and right subsequences. The position $i_{\max}$ is considered a candidate split point where the sequence may be divided, provided that the corresponding divergence, $d_{\max}\equiv d\left(i_{\max}\right)$, is statistically significant.
3. Next, assess the statistical significance of $d_{\max}$. This significance represents the probability that such a divergence could not be obtained from a random sequence $S_{0}$, i.e., the probability that the null hypothesis of a homogeneous sequence does not hold. To this end, consider the cumulative distribution function:
  (3) $$P\left(x\right)=\mathrm{Prob}\left\{\max\left[d\left(i\right)\right]\leq x\left|\;S_{0}\left(N\right)\right.\right\},$$
  which represents the probability that the maximum value of the Jensen–Shannon divergence, computed over all possible split positions, is less than or equal to *x* when segmenting a random nucleotide sequence $S_{0}$ of length *N*. For details on how to obtain P(x), see [14]. In mathematics,
  (4) $$p\left(d_{\max}\right)\equiv 1-P\left(d_{\max}\right)$$
  is called a *p*-value. It can be interpreted as the probability that the null hypothesis ($H_{0}$) is true. In our case, $H_{0}$ is that the observed $d_{\max}$ can be obtained in a sequence $S_{0}$ of random nucleotides. We reject $H_{0}$ if the *p*-value is smaller than a given threshold $p_{0}$ (usually 0.05), thus accepting the alternative hypothesis $H_{1}$ that the observed $d_{\max}$ is higher than it could be expected to occur within a random i.i.d. sequence. The acceptance of the alternative hypothesis $H_{1}$ entails the acceptance of $i_{\max}$ as a change point, i.e., the series is cut at position $i_{\max}$ into two segments. If $H_{0}$ is not rejected, the sequence remains uncut.
4. If the sequence is split, the same procedure is recursively applied to each resulting subsequence.
5. The recursion terminates when no further significant change points are detected. The sequence is then said to be segmented at a significance level of $s=1-p_{0}$. For example, if $p_{0}=0.05$, we say that the sequence *S* is segmented at 0.95 or 95% significance level.

The parameter *s* defines the statistical threshold for determining whether the difference between segments is meaningful under the null hypothesis that the sequence is random and i.i.d. or not. By adjusting this parameter, it is possible to explore the underlying distribution of segment lengths and nucleotide compositions with varying degrees of resolution [25]. This flexibility helps satisfy a central requirement of a complexity measure [26]. Using a random i.i.d. sequence as the null hypothesis effectively sets a baseline for identifying homogeneity. In essence, a sequence is considered compositionally heterogeneous—and thus in need of segmentation—when the compositional differences exceed those expected under the i.i.d. model.

In fact, based on this segmentation approach, a complexity measure was proposed in 1998 [27], and it has recently been applied to various biological systems. Notably, it was used to study the evolution of Cyanobacteria, revealing compositional shifts consistent with progressive evolution in ancient lineages [28]. It was also applied to the genome of SARS-CoV-2, where a temporal decrease in compositional complexity was observed as the virus adapted to the human host [29]. These studies highlight the utility of compositional segmentation as a powerful tool for uncovering evolutionary signals embedded within genomic sequences.

The segmentation procedure described above is computationally efficient, with a runtime proportional to O(Nlog(m−1)), where *N* is the length of the sequence and *m* is the number of resulting segments (m−1 cuts). However, it is heuristic in nature, meaning it does not guarantee identification of the optimal set of segments that fully satisfy the statistical significance criteria. As a result, segments identified as homogeneous by the algorithm may still contain internal heterogeneities. This limitation can be addressed using dynamic programming, which yields optimal algorithms with a runtime of $O(N^{2})$ [25,30]. Nevertheless, this approach is not a panacea, as it requires prior knowledge of the number of segments present in the sequence. In fact, it has been shown [31,32] that when the initial estimate of the number of segments is inaccurate, the heuristic algorithm can outperform the optimal one.

Having this in mind, the optimal segmentation algorithm is not always the best practical choice. Although it guarantees the most statistically significant partitioning of a sequence, its computational cost—proportional to $O(N^{2})$—can become prohibitive, especially when dealing with long sequences such as complete genomes. In contrast, our heuristic algorithm, while not guaranteeing optimality, has demonstrated strong practical performance in multiple genomic studies, and independent evaluations have shown that it performs remarkably well in practice [18,32]. Although the lack of optimality could lead to the presence of residual internal heterogeneities in some segments, which may slightly blur the compositional landscape, this effect is mitigated by the statistical aggregation of segment properties into histograms. Since our genome signature is based on the distribution of compositional features across many segments, rather than on individual segment boundaries, the method remains robust to minor segmentation inaccuracies. In addition, its efficiency—with a runtime of O(Nlogm)—makes it particularly suitable for large-scale genomic analyses; thus, given the size of the sequences we aim to analyze, the trade-off between computational efficiency and segmentation precision clearly favors our approach.

## 3. Compositional Landscape of the Genome

Once a genome is segmented at a significance level *s* into a set of *m* non-overlapping segments $\left\{S_{1},S_{2},\ldots ,S_{m}\right\}$, each segment $S_{i}$ can be characterized by a chosen compositional property. The distribution of the selected property across the segments defines what we refer to as the compositional landscape of the genome—a genome-wide profile that reflects how that property varies along the sequence. This landscape serves as a compact statistical representation—or signature—of the genome.

**Figure 1:**
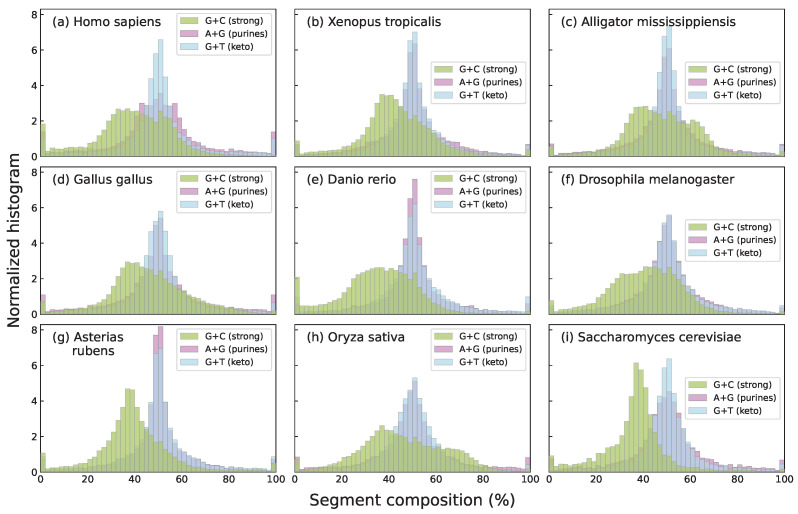
Histograms of the percentage content of strong bases (G+C), purine bases (A+G), and keto bases (G+T) for the segments obtained through segmentation at a significance level of s=0.95, applied to the complete genomes of several species across the tree of life: human (**a**), western clawed frog (**b**), American alligator (**c**), chicken (**d**), zebrafish (**e**), fruit fly (**f**), starfish (**g**), Asian rice (**h**), and baker’s yeast (**i**). Histograms were computed using 50 bins in all cases. For all three groupings the histograms reveal clear differences among species, although G+C distributions appear to be more species-specific. This species specificity of G+C histograms arises in part from the natural asymmetry in C+G content across genomes, which reflects local compositional biases and genomic architecture. In contrast, the A+G and G+T histograms are more symmetric and show less variability across species due to the approximate balance of purines and pyrimidines (A+G ≈ T+C) imposed by Chargaff’s second rule. This rule, together with base-pairing principles, also leads to an approximate balance of keto and amino bases (G+T ≈ A+C), centering both histograms around 50% and reducing species-specific variability.

**Figure 2:**
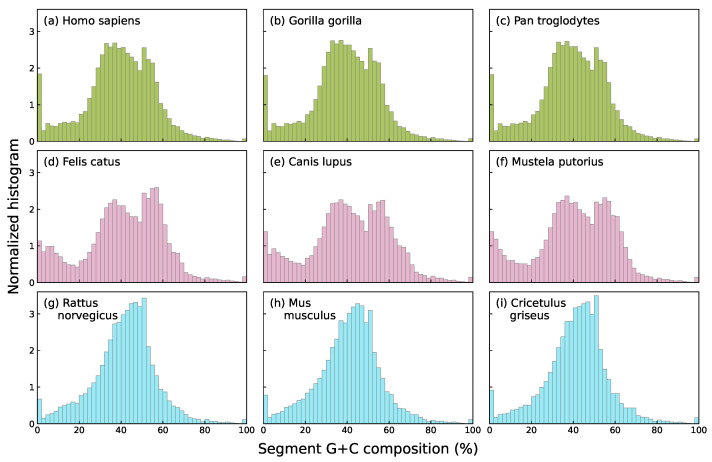
Histograms of G+C composition of the segments obtained by segmenting at s=0.95 significance level the complete genomes of three primates: human (**a**), gorilla (**b**), and chimpanzee (**c**); three carnivores: cat (**d**), dog (**e**), and polecat (**f**); and three rodents: rat (**g**), mouse (**h**), and Chinese hamster (**i**). Note that all histograms in the same row, which correspond to closely related species in terms of evolutionary divergence time [37,38], look quite similar to each other.

In this work, we focus specifically on nucleotide compositional profiles based on the skews of single-base groupings: strong/weak nucleotide ratios (GC/AT ratios, usually known as G+C content), purine/pyrimidine ratios (A+G/C+T) and keto/amino ratios (G+T/A+C), each capturing distinct chemical or structural properties of DNA sequences. Among these three, G+C content shows the strongest and most well-documented associations with key biological features, including gene density [33], codon usage bias [34], replication timing [35], and thermal stability [36]. In addition, the G+C compositional landscape tends to exhibit higher species specificity than landscapes defined by other base groupings. As an illustrative example, segmental G+C, A+G, and G+T values are shown for several species in Figure 1. The species specificity of G+C content distributions becomes even more apparent in Figure 2, which focuses on mammals and shows that species within the same taxonomic Order exhibit strikingly similar histograms.

However, it is important to note that features with strong functional associations are not always the most informative from a phylogenetic perspective. For this reason, we consider all three compositional landscapes in our analysis, rather than focusing exclusively on G+C content. In fact, as we will show later, the purine fractions yield stronger phylogenetic signals than G+C content in our framework.

## 4. Segment Compositional Distance

Having established that histograms derived from compositional segmentation are species-specific and reflect evolutionary proximity, we now seek a quantitative means of comparing them across organisms. Specifically, we aim to extract a numerical index that captures the dissimilarity between compositional landscapes—an index that could serve as a proxy for evolutionary distance or genomic relatedness, especially in contexts where traditional sequence alignment is impractical.

To this end, we reuse the Jensen–Shannon divergence (JSD), the same statistical measure employed during segmentation to detect compositional shifts within genomes. To be precise, we use its square root, which has all the properties of a metric. Here, JSD is applied at a higher level: to compare the empirical G+C, A+G, or G+T content distributions obtained from different species. This results in a distance matrix that encapsulates pairwise compositional differences, providing a compact and alignment-free representation of genomic divergence grounded in large-scale compositional structure.

To formalize this approach, we define the G+C *Segment Compositional Distance*, denoted as $D_{GC}$, as a measure of genomic dissimilarity or divergence between two organisms based on their compositional landscapes. Specifically, let us consider two genomes, indexed by *A* and *B*, each segmented at significance level *s* into compositional segments. From these segments, we construct *n*-bin normalized histograms of G+C content, denoted by probability distributions $P=(p_{1},p_{2},\ldots ,p_{n})$ and $Q=(q_{1},q_{2},\ldots ,q_{n})$, respectively, where each bin corresponds to a G+C content interval and $p_{i}$ (resp. $q_{i}$) represents the fraction of segments of genome *A* (resp. *B*) whose G+C content falls within bin *i*. These histograms satisfy

$$\sum_{i=1}^{n}p_{i}=1,\;\sum_{i=1}^{n}q_{i}=1,\;p_{i},q_{i}\geq 0\;\forall i.$$

The Shannon entropy of a discrete distribution *P* is defined as

$$H\left(P\right)=-\sum_{i=1}^{n}p_{i}\log_{2}p_{i},$$

where 0log0=0.

The Jensen–Shannon divergence between *P* and *Q* is then given by

$$d\left(P,Q\right)=H\left(\frac{P+Q}{2}\right)-\frac{1}{2}H\left(P\right)-\frac{1}{2}H\left(Q\right),$$

where the distribution (P+Q)/2 is defined component-wise as

$${\left(\frac{P+Q}{2}\right)}_{i}=\frac{p_{i}+q_{i}}{2}.$$

We define the G+C *Segment Compositional Distance* between genomes *A* and *B* as

$$D_{GC}\left(A,B\right)\equiv \sqrt{d(P,Q)}.$$

This measure is symmetric, bounded between 0 and $\log_{2}2=1$, satisfies the triangle inequality, and quantifies the dissimilarity between the G+C content compositional landscapes of the two genomes. A value of $D_{GC}\left(A,B\right)=0$ indicates identical G+C content distributions, while larger values reflect greater compositional divergence. Note that $D_{GC}\left(A,B\right)=0$ does not imply that the full DNA sequence of *A* is identical to that of *B*. The symmetry of $D_{GC}$ makes it particularly well-suited for constructing distance matrices used in comparative analyses.

The method just described for G+C content applies identically when using alternative base groupings such as A+G (purine content) or G+T (keto content), leading to the definitions of $D_{RY}$ and $D_{KM}$, respectively. In what follows, we use the generic notation D to refer to any of the three measures $D_{GC}$, $D_{RY}$, or $D_{KM}$, depending on context.

It is important to note that the Segment Compositional Distance D is robust with respect to the number of bins used in the histogram construction. Although the choice of number of bins affects the granularity of the compositional landscape, our analyses show that the overall phylogenetic signal and divergence patterns remain consistent across a wide range of binning schemes. This robustness is further supported by the results in Section 5, where phylogenetic signal metrics are shown to be stable across different numbers of bins.

## 5. Results

In Figure 2, visual inspection of the content histograms suggested that species within the same mammalian Order (primates, carnivores, or rodents) exhibit more similar compositional patterns compared to those from different groups.

**Figure 3:**
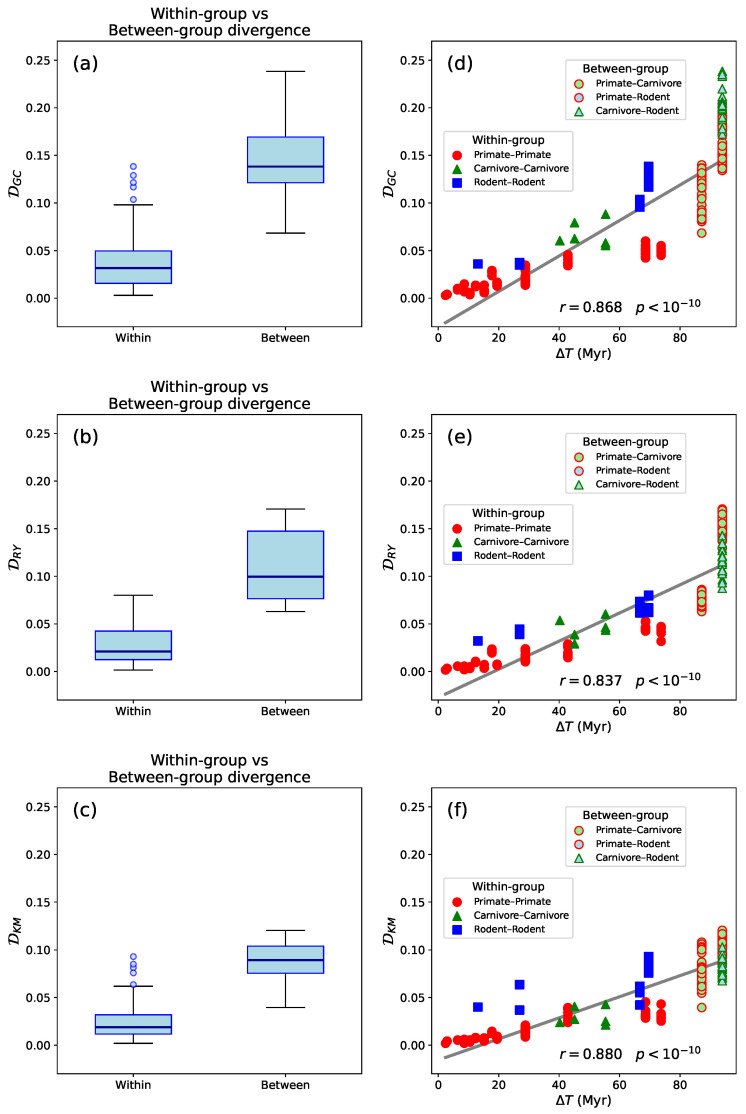
(**a**–**c**): Box-and-whisker plots showing the distributions of $D_{CG}$, $D_{RY}$, and $D_{KM}$ values, respectively, for comparisons within and between mammalian Orders. “Within-Order” comparisons include species from the same taxonomic group (primates, carnivores, or rodents), while “between-Order” comparisons involve species from different groups. Boxes represent interquartile ranges (IQR), horizontal lines indicate medians, and outliers are shown as individual points. Segment compositional distances are consistently higher for between-Order comparisons. Both Mann–Whitney *U* tests and Welch’s *t* tests yielded statistically significant differences in all cases ($p<10^{-10}$). (**d**–**f**): Relationship between segment compositional distances—$D_{CG}$, $D_{RY}$, and $D_{KM}$, respectively—and evolutionary divergence time (ΔT), defined as the estimated time since the most recent common ancestor, among the same set of mammalian species shown in Figure 2. Each point represents a pairwise comparison between species. D was calculated from segment content histograms (50 bins; significance level s=0.95). ΔT (in millions of years) was obtained from https://www.timetree.org [38] (accessed on 22 May 2025). Solid gray lines indicate linear fits to the data.

To quantitatively evaluate this observation, we computed the Segment Compositional Distance (D) for all species pairs in a representative set from the three mammalian Orders (Table 1, Figure 3a–c). Using both the Mann–Whitney *U* test and Welch’s *t*-test, we found that D values within taxonomic groups are significantly smaller than those between groups ($p<10^{-10}$). This indicates that species within the same group (primates, carnivores, or rodents) have consistently lower pairwise D values than species from different groups.

This confirms our hypothesis that D would be lower within taxonomic Orders and higher between them. This pattern is consistent with expectations from evolutionary theory and supported by previous studies [39] that describe mechanisms by which compositional differences accumulate between genomes. These include lineage-specific differences in mutation biases, DNA repair efficiency, transposable element activity, and selection on codon usage or gene regulation. Such processes shape local sequence composition over time, leading to more similar compositional patterns in closely related species due to their shared evolutionary history, and increasingly divergent patterns as phylogenetic distance increases. Hence, segment compositional distance reflects both evolutionary time and the cumulative action of genome-shaping processes.

In addition, we examined the relationship between D and divergence time, finding a strong and statistically significant positive correlation. The Pearson correlation coefficient (Figure 3d–f, r=0.868,0.837, and 0.880 for $D_{GC}$, $D_{RY}$, and $D_{KM}$, respectively with $p<10^{-10}$) indicates a strong linear association, suggesting that D increases with divergence time between species. The Spearman rank correlation coefficient, ρ=0.954,0.956, and 0.899 for $D_{GC}$, $D_{RY}$, and $D_{KM}$, respectively ($p<10^{-40}$), reveals an even stronger monotonic relationship, implying that the rank ordering of species pairs by divergence time closely mirrors their ordering by segment compositional distance. Together, these findings provides quantitative support for the time-dependent accumulation of compositional differences across mammalian genomes. Rather than merely reflecting taxonomic grouping, D appears to scale with evolutionary time, consistent with molecular clock-like behavior observed in genomic divergence rates [40]. The agreement with divergence estimates from TimeTree [37] further reinforces the reliability of this pattern. Our results also suggest that segment compositional distance is not only shaped by lineage-specific mechanisms, but also retains a measurable signature of evolutionary distance, making it a useful complement to more traditional phylogenetic markers.

Now, we extend our analysis to focus on the evolutionary trajectory of segment compositional distance from the perspective of a single species, *Homo sapiens*. Specifically, we calculated D values between the human genome and a broad set of mammalian species spanning multiple Orders and divergence times. This species-centered approach provides several advantages: first, *Homo sapiens* represents a well-annotated, high-quality reference genome commonly used in comparative genomics studies [41,42,43]; second, anchoring comparisons to a single reference enables a clearer assessment of how segment compositional distance accumulates as a function of evolutionary distance. Understanding this relationship is important to characterize the temporal dynamics of segment compositional distance, which may exhibit linear or nonlinear trends over long evolutionary timescales [39,40]. Therefore, this focused analysis complements our broader inter- and intra-Order comparisons by revealing the rate and pattern of compositional change in relation to divergence time from a fixed genomic baseline.

**Figure 4:**
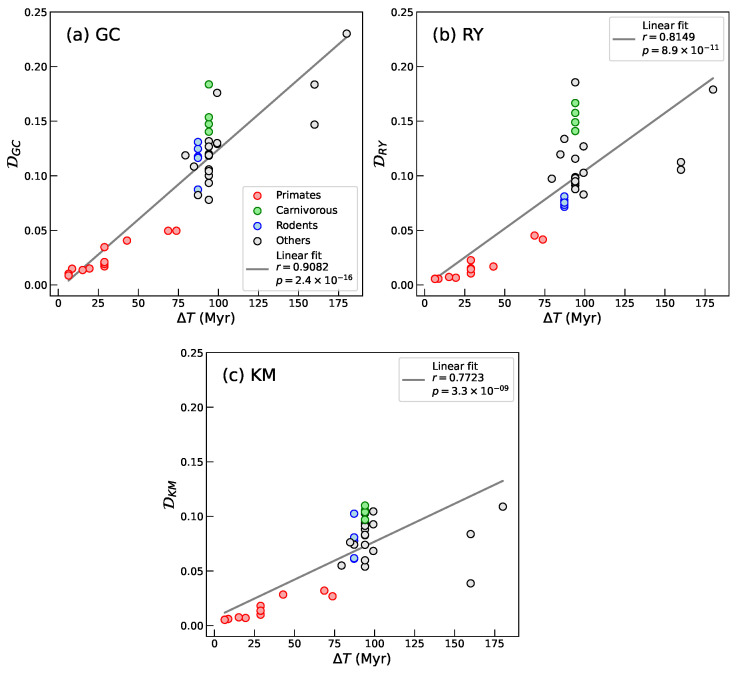
Plots of Segment Compositional Distance between *Homo sapiens* and the mammalian species listed in Table 2 from sequence segmentation at significance level s=0.95 and for 50-bin histograms, as a function of species divergence time (ΔT). (**a**) $D_{GC}$, (**b**) $D_{RY}$, and (**c**) $D_{KM}$. The solid lines in each panel represent the linear fits to the data. In all three cases we obtain strong and statistically significant lineal correlations.

We observe a strong correlation between the Segment Compositional Distance and divergence time for all nucleotide groupings—$D_{GC}$, $D_{RY}$, and $D_{KM}$ (Figure 4). This finding supports our initial hypothesis that compositional landscapes provide a robust descriptor of genome-wide divergence and are suitable candidates for capturing large-scale evolutionary trends across mammalian genomes. It further suggests that D carries a phylogenetic signal and can serve as a quantitative proxy for evolutionary distance.

Phylogenetic signal is the tendency for closely related species to display similar trait values (i.e., a specific characteristic or feature of an organism) as a consequence of their phylogenetic proximity [44]. To determine the phylogenetic signal of Segment Compositional Distance, we used three symmetric matrices of pairwise distances of the species listed in Table 2, including *Homo sapiens*, corresponding to $D_{GC}$, $D_{RY}$, and $D_{KM}$. These matrices reflect the entire compositional divergence between genomes, corresponding to strong/weak, purine–pyrimidine and keto–amino skews, respectively. Divergence times were incorporated from a calibrated, ultrametric phylogenetic tree downloaded from https://www.timetree.org [37,38] (accessed on 22 May 2025).

To obtain the phylogenetic signal, we first need to reduce each distance matrix to a trait vector that assigns a single number to each species. In doing so, we used Multidimensional Scaling (MDS), thus reducing each distance matrix to a single axis [45,46]. Finally, we used the phylosignal R package [44] to compute five indexes of phylogenetic signal (Table 3): Abouheif’s $C_{mean}$ [47], Moran’s *I* index [48,49], Blomberg’s *K* and $K^{*}$ [50,51], and Page’s λ [52].

Table 3 shows significant values for all the indexes of phylogenetic signal, save for Moran’s *I* in some cases. It is noteworthy that the phylogenetic signal index values were higher on average for $D_{RY}$ than for $D_{GC}$, indicating greater functional constraints for GC nucleotide grouping. This is consistent with the known biological significance of spatial variations in GC composition across the genome [16,53,54,55,56,57,58]. Overall, these results indicate that D exhibits a strong phylogenetic signal, making it a meaningful distance measure for studying the evolution of genome compositional structure. This result remain fairly consistent across different numbers of bins, slightly increasing the values of all indexes for a higher number of bins. However, choosing a large number of bins may not be appropriate in all cases, particularly when applying this measure to a genome composed of a small number of segments. In such cases, using too many bins could result in sparsely filled histograms and unreliable estimates of compositional distances.

To further evaluate the biological relevance of the Segment Compositional Distance, we constructed a phylogenetic tree using the distance values as input. The goal of this analysis is to assess whether the proposed measure captures meaningful evolutionary relationships among species, beyond simple pairwise correlations. This is typically done using a phylogenetic tree—a branching diagram that represents the inferred evolutionary relationships among a set of organisms, based on genetic or genomic similarity.

**Figure 5:**
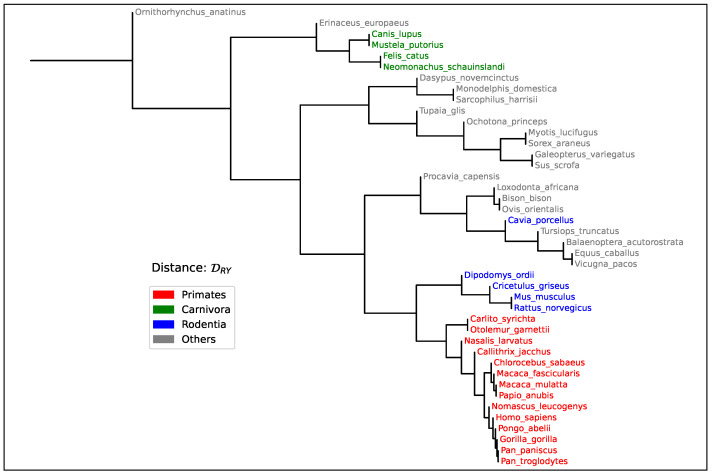
Phylogenetic tree constructed using the Segment Compositional Distance among all mammalian species listed in Table 2. Pairwise distances were computed from 50-bin histograms of Purine content ($D_{RY}$), obtained through significance-based sequence segmentation (s=0.95). The resulting topology reveals coherent taxonomic groupings (see main text), supporting the ability of the Segment Compositional Distance to reflect evolutionary relationships based on large-scale genome composition.

A well-structured tree that reflects established taxonomic groupings indicates that the distance metric encodes a robust phylogenetic signal. This analysis, conducted on the same group of mammalian species listed in Table 2, is presented in Figure 5. The resulting tree reveals several biologically consistent groupings: all primates cluster together, as do all carnivores, while rodents form a cluster with the notable exception of *Cavia porcellus* (Guinea pig), which appears separated from the main rodent clade. Interestingly, this divergence corresponds to its relatively early evolutionary split from other rodent lineages in the dataset. These observations support the utility of the Segment Compositional Distance in capturing large-scale evolutionary patterns across mammalian genomes.

## 6. Discussion

Our analyses across a representative set of mammalian genomes (Table 2) reveal that D correlates strongly with divergence time (Figure 4) and that D carries a strong phylogenetic signal (Table 3). These findings demonstrate that our method recovers a phylogenetic signal that reflects evolutionary relationships with high consistency, without relying on sequence alignment or gene annotation.

This result is particularly relevant in the context of the long-standing debate over the molecular clock hypothesis. Originally proposed by Zuckerkandl and Pauling in the 1960s [59], the molecular clock posits that genetic changes accumulate at an approximately constant rate over time. However, subsequent work has shown that evolutionary rates vary substantially across genes, lineages, and genomic regions due to differences in mutation rates, selective pressures, generation times, and DNA repair mechanisms [60,61,62]. As a result, the concept of a universal clock has largely been replaced by models that incorporate rate heterogeneity, such as relaxed clock models in Bayesian phylogenetics [63].

Our approach provides an alternative perspective: while we do not assume a constant substitution rate, the segment compositional distance still exhibits a strong, approximately linear relationship with divergence time. This suggests that the large-scale compositional structure of the genome evolves in a statistically regular manner over long timescales, despite local heterogeneities. Because D is derived from genome-wide features rather than individual mutations, it inherently averages over localized rate variation and is less sensitive to the stochasticity that affects gene-level analyses. In this sense, our method recovers a “relaxed-clock” [63] behavior without requiring explicit modeling of rate variation. Thus, our method can be interpreted as a coarse-graining of the evolutionary process, capturing stable, long-term trends in genomic composition that go beyond local rate fluctuations.

Furthermore, the observed phylogenetic consistency in trees constructed from pairwise $D_{RY}$ values (Figure 5) reinforces the notion that segmental composition reflects deep evolutionary history. An interesting observation in our phylogenetic analysis is the placement of *Cavia porcellus* outside the main rodent cluster. The fact that this pattern aligns with their early divergence from other rodent lineages may indicate a genuine evolutionary signal rather than a methodological artifact. However, this intriguing pattern could also be influenced by the faster evolutionary rates commonly observed in rodents. Duret and Galtier [64] showed that rodents tend to accumulate substitutions more rapidly than other mammalian Orders, which has been attributed to shorter generation times, higher metabolic rates, and larger effective population sizes. These factors can result in accelerated genome-wide changes, including shifts in base composition, and may cause compositional distances such as D to increase disproportionately over time. Because D captures large-scale divergence in the nucleotide groupings content landscape rather than specific substitutions, it may be especially sensitive to such rate effects when applied to lineages with unusual compositional dynamics. In particular, *Cavia porcellus* has been reported to have a highly rearranged and compositionally atypical genome [65,66], which may further accentuate its distance from other rodents in an alignment-free framework. Although our method does not rely on substitution models or assume a molecular clock, these results highlight the importance of considering lineage-specific evolutionary dynamics when interpreting phylogenetic signal from compositional data.

It is worth noting that the results presented here are practically unaffected by the specific choice of the number of histogram bins or the segmentation significance level. While the paper explicitly demonstrates the consistency of the phylogenetic signal across different binning schemes (see Table 3), similar robustness was observed for a range of segmentation thresholds.

From its definition, it follows that D requires only raw genomic sequences and operates without the need for sequence alignment. This makes it applicable to fragmented assemblies or highly divergent genomes where traditional phylogenetic methods may fail. Although high-quality genome assemblies naturally yield more reliable results, the method performs reasonably well even with incomplete or lower-quality sequences. To illustrate this, we applied D to three human genome assemblies of varying quality: GRCh37.p13 (low quality), GRCh38.p14 (medium quality), and T2T-CHM13v2.0 (telomere-to-telomere, high quality). In all cases, the distances between these assemblies were significantly smaller than the distances between T2T-CHM13v2.0 and the closest non-human primates. This confirms that while optimal results are achieved with high-quality assemblies (without annotations), D remains stable and informative across a range of sequencing qualities. This, together with its robustness, makes D a promising tool for detecting evolutionary patterns based on genome-wide compositional structure.

## 7. Conclusions

We have introduced a novel alignment-free method for quantifying genome divergence based on the segment compositional landscape of DNA sequences. This approach involves entropic segmentation of genomic sequences into compositionally homogeneous domains, followed by the construction of species-specific histograms of nucleotide groupings content. The dissimilarity between these histograms, measured using the Jensen–Shannon distance (the square root of the Jensen–Shannon divergence), defines the Segment Compositional Distance (D), a symmetric and robust distance that captures large-scale genomic structure will all desirable properties of a measure of genomic divergence.

Our analyses across a wide range of mammalian genomes demonstrate that this measure captures biologically meaningful patterns of evolutionary divergence. We observed a strong correlation between D and divergence time, both across and within taxonomic Orders, and also find that the measure retains a clear phylogenetic signal. Furthermore, phylogenetic trees constructed from pairwise distances based on D reveal coherent taxonomic groupings consistent with established evolutionary relationships. Although it might seem intuitive that closely related organisms exhibit similar genomic compositions, our method provides a structured, quantitative framework to capture and compare how compositional features (e.g., G+C or purine content) are distributed across the genome. This is not a trivial observation: similarity in nucleotide content distributions reflects not only sequence similarity, but also higher-order genomic organization that may not be evident through direct sequence comparison.

Apart from the usefulness of the genome signature D as a measure of distance between genomes, the analysis of compositional landscapes itself enables the identification of regions with distinct nucleotide usage, which may correspond to functional domains, horizontal gene transfer events, or evolutionary signatures. This approach can support genome annotation, the detection of genomic islands, and the exploration of DNA structural organization across species. By providing a scalable and statistically grounded framework, our method contributes to the broader effort of interpreting genomic complexity and variability in both model and non-model organisms.

Taken together, our results establish the Segment Compositional Distance as a computationally efficient and biologically meaningful tool for large-scale comparative genomics. It is particularly well-suited for phylogenetic analysis in cases where sequence alignment is unreliable or infeasible, and may also be useful in the study of genome evolution, taxonomy, and biodiversity through a compositional lens.

## 8. Material and Methods

- Genome sequences used in this study were retrieved from the National Center for Biotechnology Information (NCBI) Genome database, a public repository for genome data https://www.ncbi.nlm.nih.gov/datasets/genome (accessed during March and April 2025). We navigated to the “Eukaryotes” section and then filtered by “Mammalia” to find links to the various available genome assemblies.
- Implementation details, source code, and pre-compiled binaries of the segmentation program are available at https://github.com/idedis/scc (accessed on 22 May 2025).
- The Python scripts, wrapper code for the scc executable, histograms, matrices of Segment Compositional Distance, and time divergence between species (retrieved from https://www.timetree.org (accessed on 22 May 2025)) are openly available at https://github.com/idedis/genome-divergence (accessed on 22 May 2025).
- All graphs in this article were produced using Python’s Matplotlib library (ver. 3.10.3). Phylogenetic trees were visualized with the Bio.Phylo module (ver. 1.8.0) from Biopython (ver. 1.85), which integrates with Matplotlib for tree rendering. Statistical calculations and clustering procedures were carried out using Python’s SciPy library (ver. 1.15.3).
- To perform multidimensional scaling (MDS) and evaluate phylogenetic signals in our data, we used the statistical computing environment R (ver. 4.3.4) along with several dedicated phylogenetic packages. Specifically, we employed the libraries ape (ver. 5.8.1), phytools (ver. 2.4.4), geiger (ver. 2.0.11), and phylobase (ver. 0.8.12) for phylogenetic data handling and manipulation. The phylosignal (ver. 1.3.1) package was used to compute various indices of phylogenetic signal, including Abouheif’s $C_{mean}$, Moran’s *I*, Blomberg’s *K* and $K^{*}$, and Pagel’s λ, providing a quantitative assessment of trait similarity as a function of phylogenetic relatedness.
- To accelerate the segmentation and computation of genome histograms, we employed the application GNU Parallel [67] (ver. 20231122), which enabled parallel execution of tasks.
- The authors used AI-assisted tools (ChatGPT, OpenAI) to help refine the English in parts of the manuscript.

## Figures and Tables

**Table 1:** Example species from three mammalian Orders (primates, carnivores, and rodents) used to illustrate that Segment Compositional Distance (D) values between species of different Orders is greater than D values within the same Order.

Primates	Carnivores	Rodents
*Callithrix jacchus*	*Canis lupus*	*Cavia porcellus*
*Carlito syrichta*	*Felis catus*	*Cricetulus griseus*
*Chlorocebus sabaeus*	*Mustela putorius*	*Dipodomys ordii*
*Gorilla gorilla*	*Neomonachus schauinslandi*	*Mus musculus*
*Homo sapiens*		*Rattus norvegicus*
*Macaca fascicularis*		
*Macaca mulatta*		
*Nasalis larvatus*		
*Nomascus leucogenys*		
*Otolemur garnettii*		
*Pan paniscus*		
*Pan troglodytes*		
*Papio anubis*		
*Pongo abelii*		

**Table 2:** Divergence time (ΔT) between *Homo sapiens* and other mammalian species obtained from https://www.timetree.org (accessed on 22 May 2025). [38].

Scientific Name (Common Name)	ΔT (My)	Order
*Balaenoptera acutorostrata* (Minke whale)	94	Cetartiodactyla
*Bison bison* (American bison)	94	Cetartiodactyla
*Callithrix jacchus* (Common marmoset)	42	Primates
*Canis lupus* (Gray wolf)	94	Carnivora
*Carlito syrichta* (Philippine tarsier)	68	Primates
*Cavia porcellus* (Guinea pig)	87	Rodentia
*Chlorocebus sabaeus* (Green monkey)	28	Primates
*Cricetulus griseus* (Chinese hamster)	87	Rodentia
*Dasypus novemcinctus* (Nine-banded armadillo)	99	Cingulata
*Dipodomys ordii* (Ord’s kangaroo rat)	87	Rodentia
*Equus caballus* (Horse)	94	Perissodactyla
*Erinaceus europaeus* (European hedgehog)	94	Eulipotyphla
*Felis catus* (Cat)	94	Carnivora
*Galeopterus variegatus* (Sunda flying lemur)	79	Dermoptera
*Gorilla gorilla* (Gorilla)	8	Primates
*Loxodonta africana* (African elephant)	99	Proboscidea
*Macaca fascicularis* (Crab-eating macaque)	28	Primates
*Macaca mulatta* (Rhesus macaque)	28	Primates
*Monodelphis domestica* (Gray short-tailed opossum)	160	Didelphimorphia
*Mus musculus* (House mouse)	87	Rodentia
*Mustela putorius* (Ferret)	94	Carnivora
*Myotis lucifugus* (Little brown bat)	94	Chiroptera
*Nasalis larvatus* (Proboscis monkey)	28	Primates
*Neomonachus schauinslandi* (Hawaiian monk seal)	94	Carnivora
*Nomascus leucogenys* (Northern white-cheeked gibbon)	19	Primates
*Ochotona princeps* (American pika)	87	Lagomorpha
*Ornithorhynchus anatinus* (Platypus)	180	Monotremata
*Otolemur garnettii* (Small-eared galago)	73	Primates
*Ovis orientalis* (Mouflon)	94	Cetartiodactyla
*Pan paniscus* (Bonobo)	6	Primates
*Pan troglodytes* (Chimpanzee)	6	Primates
*Papio anubis* (Olive baboon)	28	Primates
*Pongo abelii* (Sumatran orangutan)	15	Primates
*Procavia capensis* (Rock hyrax)	99	Hyracoidea
*Rattus norvegicus* (Norway rat)	87	Rodentia
*Sarcophilus harrisii* (Tasmanian devil)	160	Dasyuromorphia
*Sorex araneus* (Common shrew)	94	Eulipotyphla
*Sus scrofa* (Pig)	94	Cetartiodactyla
*Tupaia glis* (Tree shrew)	84	Scandentia
*Tursiops truncatus* (Bottlenose dolphin)	94	Cetartiodactyla
*Vicugna pacos* (Alpaca)	94	Cetartiodactyla

**Table 3:** Phylogenetic signal statistics of Segment Compositional Distance for the set of mammals listed in Table 2, including *Homo sapiens*. We computed five indices for each nucleotide grouping ($D_{GC}$, $D_{RY}$, and $D_{KM}$), obtained from segmentations at s=0.95 and across different values of the numbers of bins used in the discretization of the histograms.

# of Bins	D	Abouheif’s $C_{\mathrm{mean}}$	Moran’s *I*	Blomberg *K*	Blomberg $K^{*}$	Pagel’s λ
	$D_{GC}$	0.5774 ***	NS	1.7055 ***	1.6440 ***	1.0416 ***
50	$D_{RY}$	0.5751 ***	0.0848 ***	2.0579 ***	1.8757 ***	1.0420 ***
	$D_{KM}$	0.2295 *	NS	1.3052 ***	1.2795 ***	1.0418 ***
	$D_{GC}$	0.5779 ***	NS	1.7018 ***	1.6450 ***	1.0416 ***
100	$D_{RY}$	0.5903 ***	0.0906 ***	2.1290 ***	1.9236 ***	1.0420 ***
	$D_{KM}$	0.2448 **	NS	1.3141 ***	1.2971 ***	1.0418 ***
	$D_{GC}$	0.5799 ***	0.0073 *	1.7010 ***	1.6473 ***	1.0416 ***
200	$D_{RY}$	0.6025 ***	0.0952 ***	2.1831 ***	1.9656 ***	1.0420 ***
	$D_{KM}$	0.2543 *	NS	1.3192 ***	1.3073 ***	1.0418 ***
	$D_{GC}$	0.5831 ***	NS	1.6993 ***	1.6500 ***	1.0416 ***
500	$D_{RY}$	0.6213 ***	0.0988 ***	2.2255 ***	2.0161 ***	1.0420 ***
	$D_{KM}$	0.2730 **	0.0098 *	1.3332 ***	1.3266 ***	1.0418 ***

***p<0.001; **0.001<p<0.01; *0.01<p<0.05; NS p>0.05.

## Data Availability

The original contributions presented in this study are included in the article. Further inquiries can be directed to the corresponding author.
